# A Comparative Study of the Semiconductor Behavior of Organic Thin Films: TCNQ-Doped Cobalt Phthalocyanine and Cobalt Octaethylporphyrin

**DOI:** 10.3390/molecules25245800

**Published:** 2020-12-09

**Authors:** María Elena Sánchez-Vergara, Citlalli Rios, Omar Jiménez-Sandoval, Roberto Salcedo

**Affiliations:** 1 Engineering Department, Universidad Anáhuac México, Avenida Universidad Anáhuac 46, Col. Lomas Anáhuac, Huixquilucan, Estado de México 52786, Mexico; 2 Instituto de Investigaciones en Materiales, Universidad Nacional Autónoma de México, Circuito Exterior s/n, Ciudad Universitaria, Coyoacán, Ciudad de México 04510, Mexico; citriogo@yahoo.com.mx (C.R.); salcevitch@gmail.com (R.S.); 3 Centro de Investigación y de Estudios Avanzados del Instituto Politécnico Nacional, Unidad Querétaro, Libramiento Norponiente 2000, Fracc. Real de Juriquilla, Querétaro 76230, Mexico; ojimenez@cinvestav.mx

**Keywords:** doped semiconductor, DFT calculations, flexible film, optical properties, electrical properties

## Abstract

The structure formed by cobalt phthalocyanine (CoPc) and cobalt octaethylporphyrin (CoOEP) with electron-acceptor tetracyano-π-quinodimethane (TCNQ), was studied by Density Functional Theory (DFT) methods. According to theoretical calculations, both cobalt systems can establish dispersion forces related to TCNQ and also in both cases the link between them is built by means of hydrogen bonds. Based on the results of these DFT calculations, we developed experimental work: the organic semiconductors were doped, and the thermal evaporation technique was used to prepare semiconductor thin films of such compounds. The structure of the films was studied by FTIR and Raman spectroscopy. The optical properties of the CoPc-TCNQ and CoOEP-TCNQ films were investigated by means of UV-Vis measurements. The results obtained were used to estimate the type of transitions and the optical bandgap. The results were compared to the previously calculated theoretical bandgap. The CoOEP-TCNQ film presented the smallest theoretical and experimental bandgap. Finally, the electrical properties of the organic semiconductors were evaluated from a PET (polyethylene terephthalate)/indium tin oxide (ITO)/cobalt macrocycle-TCNQ/silver (Ag) device we prepared. The CoOEP-TCNQ-based device showed an ohmic behavior. The device manufactured from CoPc-TCNQ also showed an ohmic behavior at low voltages, but significantly changed to SCLC (space-charge limited conductivity) at high voltage values.

## 1. Introduction

The demand for materials that are cheap, flexible and versatile has been growing in the ever-evolving electronics industry, which has led to a significant investment of resources in the development of organic molecular thin film technology, for use in optoelectronic devices [1,2,3,4,5]. Thin film technology is the basis of an astounding development in solid state electronics [6]. Film thickness, lattice dimensions, purity, surface roughness and the imperfection level of the layer represent significant parameters for defining the type, mechanism and stability of electrical transport [7]. Phthalocyanines (Pcs) and porphyrins have been incorporated into organic thin film transistors (OTFTs), organic photovoltaics (OPVs), and organic light emitting diodes (OLEDs). Pcs and porphyrins are a promising class of molecules that have been studied extensively, as the active material in organic electronics [8,9,10]. Such extended π-conjugation sensitizers (42π electrons for Pcs and 26π electrons for porphyrins) are particularly promising due to their efficient photoinduced electron-transfer and high absorption coefficients in the red region of the solar spectrum [11,12]. Both types of molecules offer an additional method for tuning optoelectronic properties and stacking in solid state: the replacement of the two protons in the molecule cavity with a metal ion [13] and synthetic modifications by incorporating functional groups at the outer positions of the cavity [14]. Pc and porphyrin molecules act as building blocks for diverse well-defined supramolecular nanostructures; however, structural deformations occur under external stimuli. Thus, it is particularly important to select an appropriate stimulus, when inducing alteration in terms of molecular conformations, symmetry, optoelectronic properties and crystalline phase transitions [15,16,17,18,19]. In this context, voltage is one of the most significant stimuli, as this influences electronic properties instantaneously and enhances generation of the current in device technology [20,21,22,23]. Another feature that alters electronic properties is the electron-donating or electron-accepting function performed by the Pc and the porphyrin, which form active layers inside optoelectronic devices. These molecules can act as either donors or acceptors, depending on the electron-donating or -accepting characteristics of the bound substituents, the metal center and the frontier orbital energies of the other active layer species [13,14,24,25,26,27,28].

Although remarkable progress has been made for porphyrin-based optoelectronic devices, the low charge transport properties of porphyrin and its derivatives significantly limit the efficient charge transport in films for further improvement of device performance [13,14,29,30,31,32,33,34,35]. In contrast, Pcs exhibit excellent charge transport characteristics, as well as a widely tunable absorption wavelength region [14,29]. Another advantage of Pcs, compared to porphyrins, is their generally longer exciton diffusion and higher hole mobilities [13,30,31,32,33,34,35]. Although over 70 metallic and nonmetallic ions will fit into the phthalocyanine cavity [13], Cu and Zn have been the most common choices to date for use in optoelectronic devices [13]. This is why it is interesting to study the effect of other metallic ions, such as cobalt, and compare their optoelectronic behavior with analogous porphyrin complexes. Cobalt(II) phthalocyanine (CoPc) derivatives take advantage of the exceptional physical properties that this type of macrocycle exhibits [36,37,38,39]. CoPc derivatives can be seen as fundamental representatives of a potentially low-dimensional material class, where the interaction is intimately connected to a transfer of magnetic moment or spin, and which may be distinguished by interesting and unusual physical properties [37,39]. Additionally, CoPc and some derivatives containing special hexafluoroisopropanol (HFIP) substituents are used in the hybrid sensing device and fuel cell fabrication, due to their superior solubility, the selective adsorption of vapor and on account of the electron-withdrawing effect of fluorine atoms in HFIP substituents [36,37,38].

The objective of this work is to compare the effect of CoPc and CoOEP (cobalt(II) octaethylporphyrine) as dopants, when using the electronic acceptor tetracyano-π-quinodimethane (TCNQ) as host/matrix. TCNQ, as previously reported by some of the authors of this work [40,41] has interesting electrical properties, due to its low dimensionality and energy-band formation, as well as its ability to yield or accept electrons at a low energy cost, which results in a partially occupied higher energy band [40,41]. By means of DFT (density-functional theory) calculations, the possible interaction between the cobalt macrocycles and the TCNQ was obtained, as well as the HOMO-LUMO orbitals and the theoretical bandgap of each system. According to such results, these are low bandgap materials with possible optoelectronic applications, which led us to prepare thin films of disperse heterojunction with these materials. The characterization of the films was performed by means of FTIR and Raman spectroscopy. The absorption coefficient and photon energy, as well as the optical bandgap, were obtained from a UV-Vis spectroscopy analysis. The theoretical bandgap results were compared to those obtained experimentally, and since they are within the range of organic semiconductors, the electrical behavior of CoPc- and CoOEP-TCNQ thin films was evaluated as an integral part of electronic devices deposited on PET (polyethylene terephthalate). The flexible devices were manufactured by the thermal evaporation method and their behavior was compared.

## 2. Results and Discussion

### 2.1. DFT Results and Discussion

**Figure 1:**
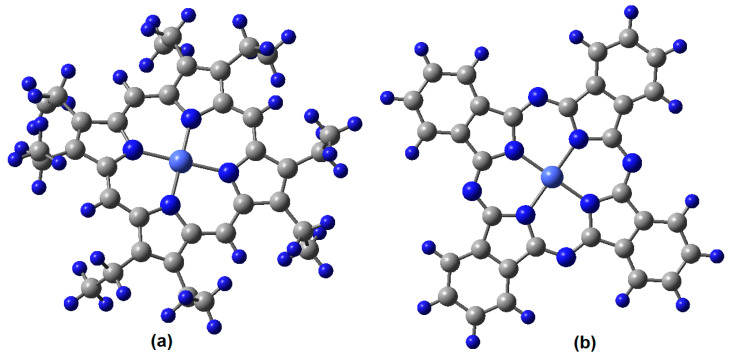
(**a**) Cobalt(II) octaethylporphyrin complex and (**b**) Cobalt(II) phtalocyanine complex.

Representations of the transition metal complexes considered in this study are shown in Figure 1. Theoretical calculations were carried out in order to establish the possibility of formation of charge transfer complexes between these molecules and TCNQ. Both cobalt systems, with phtalocyanine and porphyrin, can establish dispersion forces related to TCNQ; additionally, in both cases the link between them is built by means of hydrogen bonds, which arise from the interaction of terminal nitrogen atoms of TCNQ and peripheral hydrogen atoms from the organic rings. There are different bond lengths and positions for such interactions, since the transition metal complexes have two different kinds of peripheral hydrogen atoms: those which are joined at the end of the aromatic rings and those which saturate the methyl groups; this last category is found only in the porphyrin case. The hydrogen bond interactions can only be established on the periphery of the rings; therefore, two kinds of interactions may exist: one with hydrogen atoms with an aliphatic source and one with hydrogen atoms with an aromatic source. The description of both kinds of structures is given below in Figure 1.

**Figure 2:**
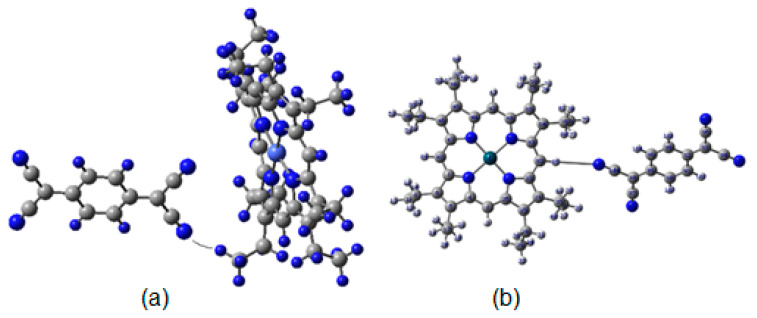
Hydrogen bond complexes between cobalt octaethylporphyrin (CoOEP) and tetracyano-π-quinodimethane (TCNQ): (**a**) hydrogen atoms with an aliphatic source and (**b**) hydrogen atoms with an aromatic source.

Firstly, there are two possible complexes between the cobalt porphyrin compound and TCNQ via hydrogen bonds, as is indicated above. They arise because the porphyrin ring has two types of terminal hydrogen atoms on its periphery. The first group corresponds to the saturation sphere of the methyl groups and the other to the sole hydrogen atom on the aromatic ring between the corner rings. Calculations indicate that both hydrogen bonds can be formed. Figure 2 shows the structure of both kinds of porphyrin complexes, specifically showing the possible hydrogen bonds. The bond lengths in the two cases vary, but both values are short enough to allow this kind of interaction. The hydrogen bond between the nitrogen atom from TCNQ and the aliphatic hydrogen atoms from the porphyrin is very short, 2.13 Å, in contrast to that with aromatic terminal hydrogen atoms of the same molecule, which is 2.94 Å. However, the Grimme correction energy difference between the two kinds of hydrogen bond is not particularly large; the first one is 10.9 kcal/mol and the second is 8.9 kcal/mol. A statistical Boltzman energy distribution analysis shows that the population of both conformations is 57%, favoring the shorter interaction structure.

**Figure 3:**
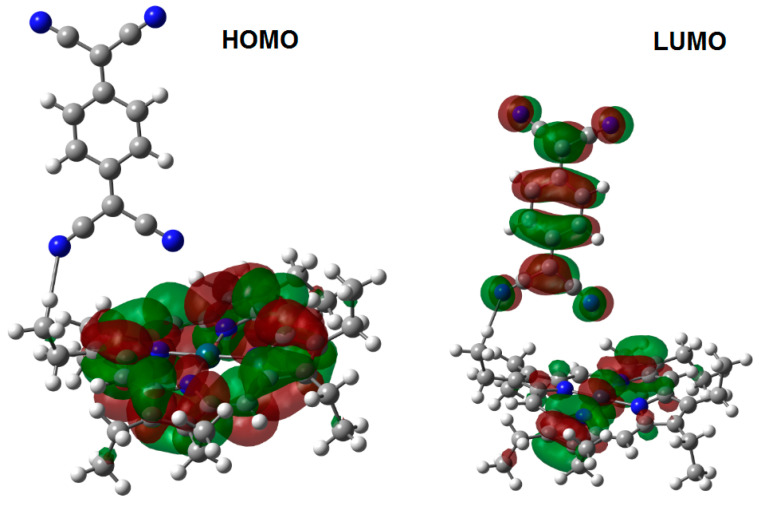
Frontier molecular orbitals of the cobalt(II) porphyrin complex with hydrogen bonds with an aliphatic source.

**Figure 4:**
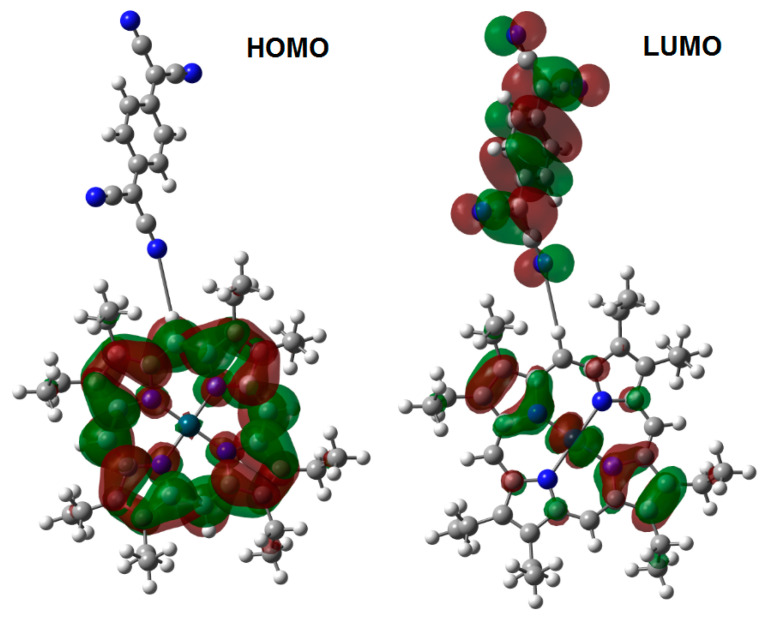
Frontier molecular orbitals of the cobalt(II) porphyrin complex with hydrogen bonds with an aromatic source.

The frontier molecular orbital analysis is in agreement with the behavior predicted for the charge transfer complexes, and Figure 3 and Figure 4 show the shape of the HOMO (Highest Occupied Molecular Orbital, π) and LUMO (Lowest Unoccupied Molecular Orbital, π*) for both conformations; however, notably in the case of the HOMO, there is an accidental degeneration, and in the case of the LUMO, the LUMO+1 orbital is close enough to the LUMO to suggest a similar accidental degeneration. As is expected for the HOMO in both cases, the strong probability of electron presence is found in the zone of the peripheral framework of the porphyrin moiety. Similarly, the LUMO shows the presence of empty regions mainly in the TCNQ zone, although there is also participation of the porphyrin ring’s central part, i.e., the cobalt atom coordination sphere. The energy gap for this species is 0.89 eV. The average value for an energy gap should be 0.9 eV, a result which suggests strong semiconductor behavior. The scheme for the second conformation is practically the same, and the band gap is 0.91 eV, meaning that the behavior should be similar in both cases, in spite of the different positions and lengths of the hydrogen bonds. Based on the above, the calculated energy value for the HOMO is of −8.245 eV, while the value for the LUMO is −7.265 eV.

**Figure 5:**
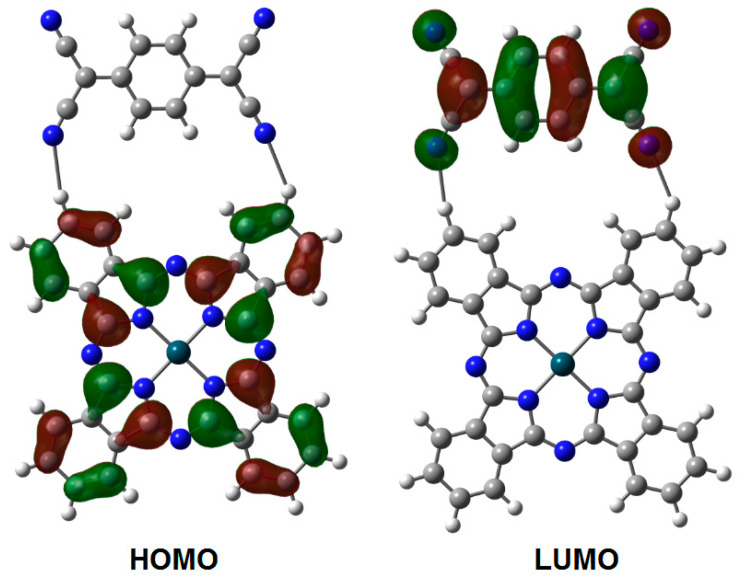
Frontier molecular orbitals of the cobalt(II) phtalocyanine complex.

In the case of the phtalocyanine complex, the TCNQ moiety settles into a horizontal position, in order to generate two interactions between itself and the lateral aromatic rings, resulting in two hydrogen bonds with an average length of 2.43 Å and a total energy of 18.5 kcal/mol. The frontier molecular orbital analysis shows that the HOMO probability is focused on the phthalocyanine moiety, whereas the LUMO is mainly found in the TCNQ zone; these probability regions are presented in Figure 5. In this case, the values calculated for the HOMO and the LUMO are −5.333 and −3.075 eV, respectively, and the band gap is 2.35 eV, which indicates a moderate semiconductor behavior. These DFT calculations are indicative of a better semiconductor behavior in the CoOEP-TCNQ system, with respect to CoPc-TCNQ. The above may be due to the greater number of interactions through hydrogen bonds and the shorter bond length of 2.13 Å between the nitrogen atom from TCNQ and the aliphatic hydrogen atoms from the porphyrin.

### 2.2. Experimental Results and Discussion

**Figure 6:**
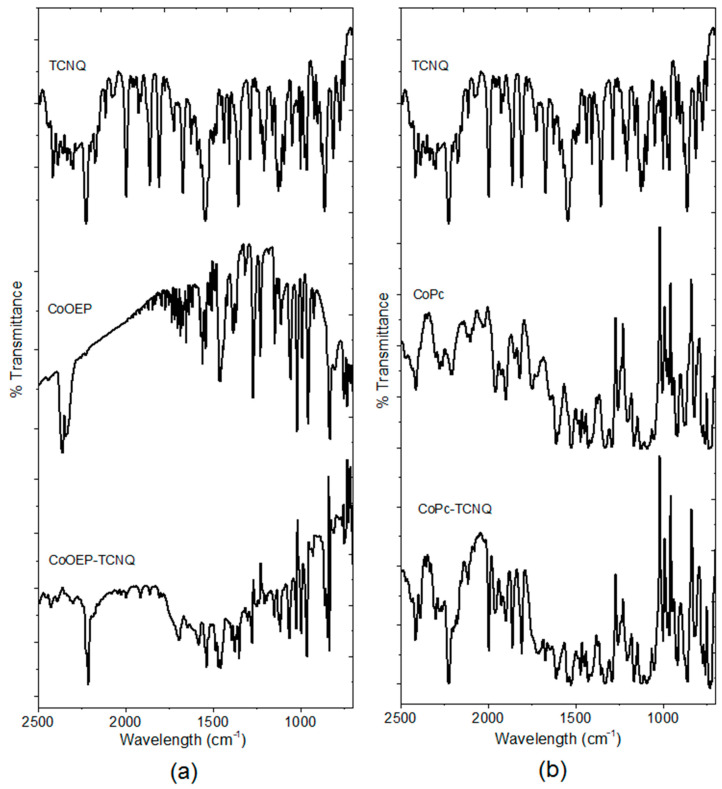
FTIR spectra of the (**a**) CoOEP-TCNQ and (**b**) CoPc-TCNQ films and their precursors.

On the basis of the DFT calculations, the preparation of the semiconductor materials CoPc-TCNQ and CoOEP-TCNQ was carried out. In this molecular doping, the mixture between the acceptor and the two donor species is carried out, in order to facilitate the electronic transfer between both types of species. TCNQ is chosen to be the host/matrix organic material and the cobalt complexes have been used as dopants. The quantity of CoPc and CoOEP in the semiconductor material was controlled in terms of their mass and in a macrocycle-TCNQ ratio of 0.3:1 [42,43]. The doping of organic semiconductors usually requires this dopant:matrix ratio, so that dopant-semiconductor and dopant-dopant intermolecular contacts are fostered [42]. In order to study the possible applications for molecular electronics, thin films of both semiconductors were deposited by the low-pressure evaporation technique. As the semiconductors are subjected to considerable thermal gradients during their evaporation and deposition, FTIR spectroscopy was employed to verify that no decomposition of these materials occurred. The FTIR spectra of the semiconductor films deposited over monocrystalline silicon were compared to those obtained for the powders as KBr pellets, and the band assignments are presented in Table 1. The FTIR spectra for the CoOEP-TCNQ and CoPc-TCNQ doped films and their precursors (TCNQ, CoOEP and CoPc) are shown in Figure 6. CoOEP (Figure 1a) is made up of cyclic tetrapyrroles; the pyrrole forms a cycle by means of bridging carbons and differs from CoPc (Figure 1b) in that the latter contains nitrogen atoms in a meso-position. CoPc presents four isoindole units (a pyrrole ring fused to a benzene ring), joined by four nitrogen atoms. The metallooctaethylporphyrine (MOEP) characteristic bands are located around 1468, 1230, 992, 924 and 755 cm^−1^ for the powder [44,45,46,47,48], which are very close to the position observed for the same bands in the films. As for the CoPc-based semiconductor, the band responsible for the pyrrole in-plane stretching vibration in the phthalocyanine ring is observed at 1332 cm^−1^ and the bands located at 1288, 1164, 1120 and 754 cm^−1^ are the result of the interaction between the carbon and hydrogen atoms [49]. The bands observed around 1611 and 1476 cm^–1^ result from a C=C stretching mode [49,50,51]. Similarly, the C≡N stretching band of TCNQ appears around 1673 cm^−1^; the peaks around 1615, 1451 and 1207 cm^−1^ are related to C=C-H bending, C-CN stretching and C=C ring stretching, respectively [52]. As is apparent in Table 1, the results of KBr pellets resemble closely those obtained for thin films. The slight changes in values are ascribed to the concentration of efforts in the bonds, due to the thermal shock between the temperature of the semiconductor in gaseous state and the room temperature of the substrates. In summary, the FTIR technique indicates that CoOEP-TCNQ and CoPc-TCNQ molecules manifest chemical stability and that the evaporation technique is favorable for obtaining films of these materials. Moreover, FTIR spectroscopy confirms the presence of the expected hydrogen bonds for the CoPc-TCNQ and CoOEP-TCNQ systems. These bonds can be indirectly detected by the displacement of the strong peak around 2225 cm^−1^, which is characteristic of the lower frequency absorbance of conjugated nitriles [53,54].

**Figure 7:**
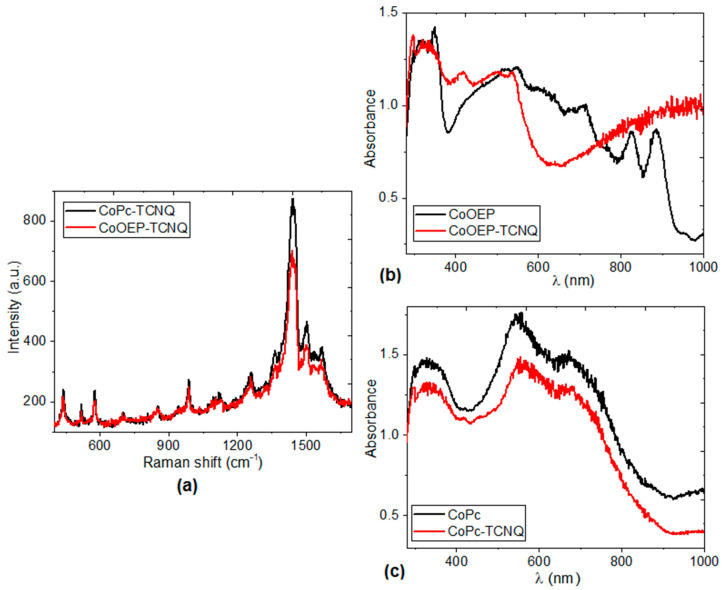
(**a**) Raman spectra of the CoPc-TCNQ and CoOEP-TCNQ thin films; UV-vis spectra of the (**b**) CoOEP-TCNQ and (**c**) CoPc-TCNQ films and their corresponding precursors CoOEP and CoPc.

The Raman spectra in Figure 7a confirmed the presence of the CoOEP or CoPc and the TCNQ moieties within the films. The peaks observed in the 400–1700 cm^−1^ range are associated with the vibrational modes in the CoOEP and CoPc molecules. Bands of benzene ring deformation appear at 589 and 592 cm^−1^, and macrocycle breathing bands are observed at 697 and 703 cm^−1^, connected to the pyrrole stretching for CoOEP and CoPc, respectively [55,56,57,58]. The major changes in the Raman spectra for the different films are observed in the range from 1200 to 1550 cm^−1^. This region corresponds to cobalt-nitrogen in-plane stretching and bending vibrations, as well as displacements on the C-N-C bridge bond of the macrocycle [55,56,57,58]. The position change of the pyrrole stretching band is observed in Figure 7a. The band changes from 1536 cm^−1^ for CoOEP to 1541 cm^−1^ for CoPc [55]. In a similar way to that found using IR spectroscopy, the presence of the TCNQ molecule is observed in the Raman spectra: 1257, 1443 and 1568 cm^−1^ signals, ascribed to C=C-H bending, C-CN stretching, C=C ring stretching, respectively [58,59]. The differences in the Raman spectra of the films originate in the macrocycle arrangement. Significantly, the presence of peaks of considerable intensity around 986, and 1363 cm^−1^ may be indicative of the molecular interactions between the CoOEP or CoPc and TCNQ [55,56,57,58,59].

The spectral distribution of absorbance, measured at nearly normal incidence in the 200–1000 nm wavelength range for the CoOEP-TCNQ and CoPc-TCNQ films and their precursors CoOEP and CoPc is shown in Figure 7b,c. The differences in the spectra are due to the presence of TCNQ. At longer wavelengths (λ > 800 nm) the CoOEP-TCNQ film becomes almost transparent in the non-absorbing region [44,60], while at shorter wavelengths (λ < 800 nm), the film presents absorption (Figure 7b). The main characteristic of the absorption zone of the CoOEP complex is the presence of two bands, the B band in the UV region of the spectrum, between 300–360 nm, and the Q band in the visible region, between 560 and 715 nm. The B and Q bands are associated with π-π* transitions, arising from the π conjugated electrons of the CoOEP structure. The Q band is associated with the π-π* transitions from the HOMO to the LUMO, corresponding to transitions from orbital a_1u_ to orbital e_g_ (see Figure 3 and Figure 4). The B band is also the result of π-π* electronic transitions, but from orbitals a_2u_ and b_2u_ to the LUMO e_g_. On the other hand, the two UV-Vis characteristic bands are observed in the spectrum of the CoPc-TCNQ film: the Q band between 548 and 709 nm, and the B band between 300 and 371 nm (Figure 7c). When comparing the spectrum of CoPc and that of the CoPc-TCNQ film, it is observed that the presence of TCNQ shifts the Q and B bands towards the ultraviolet region and also slightly decreases the absorption of the material.

**Figure 8:**
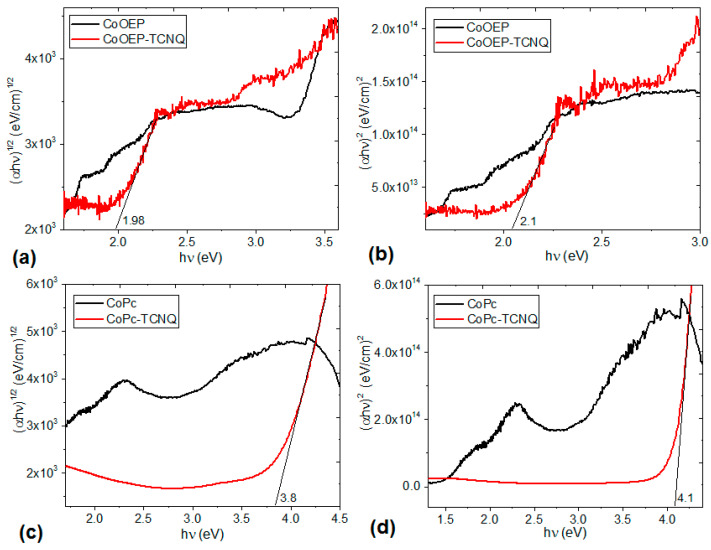
Plots of (α*hν*)^1/2^ and (α*hν*)^2^ vs. *hν* for the (**a**,**b**) CoOEP-TCNQ and (**c**,**d**) CoPc-TCNQ films and their precursors CoOEP and CoPc.

The electronic transitions in organic semiconductors can be described in terms of the band theory [44]; it is proposed that the valence band is formed by the HOMO, whereas the LUMO contributes to the conduction band [44,60,61]. In organic semiconductors the type of optical transition is usually studied, as well as the value of the optical energy gap. The absorption data conform to power-law behavior [61]:

$$\alpha h\nu \;=\;A{\left(h\nu -{E_{g}}^{\mathrm{opt}}\right)}^{r}$$

here, parameter *A* depends on transition probability, α is the absorption coefficient, E_g_^opt^ is the optical band gap and r is a number that characterizes the transition process, where r = ½ and r = 2 for direct and indirect allowed transitions, respectively [61]. The dependence of (α*hν*)^r^ on *hν* photon energy was plotted and the optical band gap was evaluated from the *x*-axis intercept at (α*hν*)^1/2^ = 0 and (α*hν*)^2^ = 0. Figure 8 shows that the CoOEP-TCNQ (Figure 8a,b) film has a greater semiconductor nature than the CoPc-TCNQ film (Figure 8c,d). Not only is the optical gap lower in the case of the film containing porphyrin, but apparently this organic semiconductor undergoes several transitions. The change in slope is an indication that more than one energy transition occurs between the energy bands of the CoOEP-TCNQ film. The first transition is the E_g_^opt^ that corresponds to the onset of optical absorption and the formation of a bound electron-hole pair, or Frenkel exciton; the second transition is the fundamental energy gap (E_g_) [60,61]. Another aspect to consider is that indirect transitions are the dominant transitions in thin films. This is due to the amorphous nature of the films, as a result of the deposit method used. Finally, when comparing the curves of the macrocycle-TCNQ systems with those of their corresponding precursors CoPc and CoOEP, marked differences are observed between them, as a result of the presence of TCNQ. For the case of the CoPc-TCNQ film, the optic band gap is greater than that of the CoPc macrocyclic complex. Apparently, the TCNQ does not enhance the charge transport in the phthalocyanine, and the CoPc-TCNQ films have a low semiconductor behavior. In the case of the CoOEP-TCNQ system, the electronic transitions are favored within the doped semiconductor. The optical band gap values obtained are within the range reported for cobalt porphyrin films [60]; however, in the present case, it was not necessary to perform an annealing treatment to reduce the band gap. It is the presence of the HOMO in the zone of the peripheral framework of the porphyrin moiety and the LUMO in the TCNQ region and the metal coordination sphere which favors the charge transport and the low bandgap observed.

**Figure 9:**
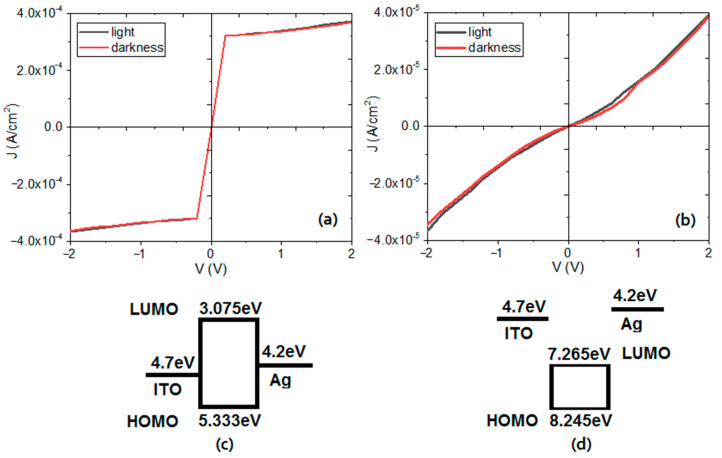
J-V plots and energetic diagrams for the (**a**,**c**) CoPc-TCNQ and (**b**,**d**) CoOEP-TCNQ flexible devices.

As expected, for both macrocycle-TCNQ films, the optical band gap obtained experimentally is higher than the bandgap calculated from DFT because the theoretical bandgap does not consider external effects of the semiconductor molecules (nature of the films, presence of impurities and degree of stacking of the electron donor and acceptor molecules). However, the same trend is observed in both the experimental and theoretical band gap results: the lowest band gap values are those for the film with CoOEP (1.98 and 2.1 eV for indirect and direct transitions, respectively), while the CoPc film shows a band gap of 3.8 eV for indirect transitions and 4.1 eV for direct transitions. These results are interesting due to the fact that Pcs normally have better charge transport properties compared to porphyrins [14,29]. It seems that when the cobalt(II) ion is at the center of the macrocycle, the behavior changes, probably influenced by the two types of hydrogen bonds that are generated between TCNQ and CoOEP (Figure 2). The electrical characterization (J-V) of the devices constructed with both kinds of materials (Figure 9a,b) complement the previous results. In both graphs, it is observed that there is no difference between the measurements made on the flexible devices in dark conditions and in natural lighting. This behavior is an indication that these films do not exhibit photovoltaic properties; however, they are candidates to be used in other types of optoelectronic devices. The CoOEP-TCNQ film shows an ohmic behavior, while the CoPc-TCNQ film also has an ohmic behavior at low voltages, but changes to a space charge limited current (SCLC) mechanism at high voltages. The electric resistor (R) for ohmic regions is calculated directly from the Ohm equation. Considering a voltage of 0.2 V, which is in the linear part of both graphs of Figure 9, the resistor is of 19.8 Ω and of 2368.57 Ω for the CoPc-TCNQ and CoOEP-TCNQ devices, respectively. These values are below those reported for the widely used CuPc [62], which is indicative of the adequate charge transport capacity of these doped films. However, the resistor in the device with CoOEP-TCNQ is two orders of magnitude higher than the resistor obtained in the phthalocyanine-based device. According to Figure 9a,b, these results are an indication that the CoOEP-based device has the behavior of a resistor, while the CoPc-based device functions like a diode. In the CoOEP-TCNQ film the current density is low, there is no excess electrical charge within the film for any applied voltage (Figure 9b,d). The film forms ohmic contacts with the ITO and Ag electrodes and there is a good energetic correlation between the work function of the electrode and the energetic level that participates in the charge transport. Furthermore, the polarity in the device is totally symmetrical, with no current rectification. When the polarity of the voltage applied across the electrodes is reversed, the current density has the same magnitude. On the other hand, the device with the CoPc-TCNQ film (Figure 9a,c) changes its behavior around 0.23 V; while at lower voltages (<0.23 V) the ohmic behavior keeps predominating, at higher voltages (>0.23 V) an SCLC transport governed by an exponential trap distribution is observed (Figure 9a) [63,64,65]. In CoPc-TCNQ, the electric transport properties are associated with the tunneling of charges through the barriers due to the difference between the HOMO and LUMO energetic levels of the semiconductor and the work function levels of the electrodes: ɸ_ITO_ = 4.7 eV and ɸ_Ag_ = 4.2 eV (Figure 9c). Since the contact is ohmic, in the vicinity of the electrodes, spatial charges are formed, which oppose the flow of current through the organic semiconductor CoPc-TCNQ; with a high enough applied field, a saturation in the current is produced.

The highest current density of 4 × 10^−4^ A/cm^2^ is obtained in the CoPc-based device. This value is one order of magnitude higher than the current density flowing through the porphyrin device. As for the density of the CoPc precursor, some of the authors of this work have reported *J*~4.75 × 10^−8^ A/cm^2^ values for CoPc and its derivatives [66,67], so apparently TCNQ favors current flow through the device. Finally, for CoPc-TCNQ in the ohmic region (Figure 9a), *J* is given by the equation [63,64]:

(1) $$J\;=\;p_{0\;}e\mu \frac{V}{d}$$

where *p*_0_ is the concentration of thermally generated holes, *e* the electronic charge, *μ* the hole mobility, *V* the applied voltage and *d* the film thickness. In the SCLC region, *J* is given by the expression [63,64]:

(2) $$J\;=\;N_{v}\;e\mu \;{\left(\frac{\varepsilon_{r}}{e\;P_{0}\;k\;T_{L}}\right)}^{l}\frac{V^{l+1}}{d^{2l+1}}\;\;\;\;\;$$

where in addition to the previously defined symbols, $N_{v}$ is the effective density of states in the valence band, $\varepsilon_{r}$ is the permittivity, *P_0_* is the concentration of traps per unit of energy, k is Boltzmann constant and $T_{L}$ is the temperature parameter which characterizes the trap distribution, and:

(3) $$l\;=\;\frac{T_{l\;}}{T}$$

where *T* is the ambient temperature. The total trap concentration *N_t(e)_* is equal to:

(4) $$N_{t\left(e\right)}\;=\;P_{0}\;k\;T_{L}$$

The electrical parameters of the CoPc-TCNQ device calculated using equation (1) for the ohmic region and equation (2) for the SCLC region are shown in Table 2. The *μ* value is in the interval reported for other devices manufactured with phthalocyanine derivatives (10^−10^–10^−7^ m^2^ V^−1^ s^−1^) [35,43,64,65]. With respect to the results obtained for the concentration of thermally generated holes (*p_0_*), they are larger than those found for NiPc and CoPc thin films and of the same order of magnitude of those reported for CuPc and ZnPc derivatives [35,63,64]. The above is considered an indication that the CoPc-TCNQ films are better semiconductors than CoPc films without dopant. As regards the concentration of traps per unit of energy (Po), the value obtained is within the range reported for some MPcs (M = Ni, Cu, Co, Pb, Zn): 8 × 10^43^ to 1.15 × 10^47^ J^−1^ m^−3^. Finally, the total trap concentration (*N_t(e)_*) calculated is also in the range of the same phthalocyanines previously mentioned (6 × 10^20^ to 9.3 × 10^26^ m^−3^) [35,43,63,64,65,66,67,68,69]. Of course, these values depend on the purity of the MPc, the doping conditions, the phases present, the thermal treatment and the material of the electrodes in the devices. In the current study it is evident that, although the CoOEP-TCNQ system exhibits a lower theoretical and experimental band gap, as well as two types of interactions through hydrogen bonding, it is the device with CoPc-TCNQ which presents a better electric behavior. According to the diagram of Figure 9c, the energy position of the HOMO and LUMO orbitals respect to the work function of the electrodes gains importance in the electric charge flow.

## 3. DFT Calculations

All calculations were carried out by applying a DFT method based on a combination of Becke’s gradient corrections [70] for exchange and Perdew-Wang’s for correlation [71]. This is the scheme for the B3PW91 method, which is included in the Gaussian16 [72] package. The calculations were performed using the 6-31G** basis set. Frequency calculations were carried out at the same level of theory in order to confirm that the optimized structures were at a minimum of the potential surfaces. The hydrogen bonds were studied by applying the Atoms in Molecules Theory (AIM) [73] and the dispersion Grimme correction (G3), taking advantage of the version included in the DFT-D3 method [74].

## 4. Materials and Methods

CoPc (cobalt(II) phthalocyanine: C_32_H_16_N_8_Co), CoOEP (2,3,7,8,12,13,17,18-octaethyl-21*H*,23*H*-porphine cobalt(II): C_36_H_44_CoN_4_) and TCNQ (7,7,8,8-tetracyanoquinodimethane: C_12_H_4_N_4_) were purchased from Sigma-Aldrich (Saint Louis, MO, USA) and required no further purification. The doped organic semiconductors were synthesized in a heated Monowave 50 reactor (Anton Paar México, S.A. de C.V., Hidalgo, Mexico), which was operated using a borosilicate glass vial with an integrated pressure (0–20 bar) and temperature sensor. A total of 1.03 g (5 mmol) of TCNQ was added to 1 g (1.7 mmol) of CoPc or 1 g (1.7 mmol) of CoOEP and dissolved in methanol or propanol, respectively. They were kept in the reactor for 25 min and then cooled and brought back to atmospheric pressure; next, they were filtered, washed and dried in vacuum. The doped semiconductors were subsequently deposited by the vacuum evaporation technique (Intercovamex, S.A. de C.V., Cuernavaca, Morelos, Mexico) onto different substrates: monocrystalline n-type silicon wafers (c-Si), quartz and indium tin oxide (In_2_O_3_·(SnO_2_)_x_) coated polyethylene terephthalate (PET-ITO) films. Previously, all substrates, excluding PET-ITO, were cleansed by applying an ultrasonic process, using chloroform, methanol and acetone, and then dried in vacuum. Using tantalum crucibles, the doped semiconductors were heated to 580 K in order to produce their phase change, which was initially carried out in the gaseous state, so that they would finally be deposited in the form of thin films upon contact with the substrates, which were held at room temperature and a vacuum pressure of 1 × 10^−6^ Torr. Due to the different melting temperatures of each doped semiconductor, the deposit speed was 0.8 Å/s. The thickness of each layer was monitored using a microbalance quartz crystal monitor, connected to a thickness sensor.

To verify the main functional groups of the organic ligands, an FTIR spectroscopy analysis was performed for the compounds as KBr pellets and for the films on silicon substrates, using a Nicolet iS5-FT spectrometer (Thermo Fisher Scientific Inc., Waltham, MA, USA) at a wavelength range of 4000 to 500 cm^−1^. The Raman spectra of the films on quartz substrates were recorded on a Horiba LabRAM HR Evolution spectrometer (Horiba, Ltd., Minami-ku Kyoto, Japan), using the 632.8 (2 mW) nm line of an HeNe laser for spectral excitation and a 50× objective. The absorbance and transmittance of the powders and films on quartz were obtained in the 200–1100 nm wavelength range, on a UV-Vis 300 Unicam spectrophotometer (Thermo Fisher Scientific Inc., Waltham, MA, USA). For the electrical characterization of the devices on ITO-coated PET substrates, a programmable voltage source, a sensing station with lighting and temperature controller circuit from Next Robotix (Comercializadora K Mox, S.A. de C.V., Mexico City, Mexico) and an auto-ranging Keithley 4200-SCS-PK1 pico-ammeter (Tektronix Inc., Beaverton, OR, USA) were employed. The evaluation of the electrical behavior of the flexible devices was performed both under lighting conditions and in darkness. The film sequence PET/ITO/cobalt macrocycle-TCNQ/Ag was used in the device setup. After manufacturing the devices, their electric properties were studied to carry out the current density vs. voltage analysis and to determine the type of electric behavior in the devices.

## 5. Conclusions

According to the DFT theoretical calculations, the cobalt macrocycles bind to TCNQ by hydrogen bonds. There are two possible complexes between the CoOEP and TCNQ: the first corresponds to the saturation sphere of the methyl groups and the other to the sole hydrogen atom on the aromatic ring between the corner rings. The hydrogen bond length in the first case is 2.13 Å, while in the second possible complex is 2.43 Å. In the case of the phtalocyanine complex, the TCNQ settles into a horizontal position, in order to generate two interactions between itself and the lateral aromatic rings, resulting in two hydrogen bonds with an average length of 2.43 Å. Experimentally, we synthesized the TCNQ doped semiconductors and prepared thin films by the thermal evaporation technique. The CoOEP-TCNQ system exhibits the smallest theoretical and experimental band gap (0.89 and 1.98 eV, respectively), which is an indication of its better semiconductor behavior. In additional work, flexible PET/ITO/cobalt macrocycle-TCNQ/Ag devices were manufactured, and upon evaluating their electrical characteristics (J-V), it was found that the CoOEP-TCNQ film has the behavior of a resistor, while the CoPc-TCNQ film shows an ohmic behavior at low voltages, which significantly changed to SCLC at high voltage values. The highest current density of 4 × 10^−4^ A/cm^2^ is obtained for the CoPc-based device. This value is one order of magnitude higher than the 4 × 10^−5^ A/cm^2^ flowing through the porphyrin device.

## Figures and Tables

**Table 1:** FTIR band positions and assignments of CoOEP-TCNQ and CoPc-TCNQ as obtained (powders) and as thin films.

CoOEP-TCNQ Assignment	Powder as KBr Pellet (cm^−1^)	As-Deposited Films (cm^−1^)	CoPc-TCNQ Assignment	Powder as KBr Pellet (cm^−1^)	As-Deposited Films (cm^−1^)
δ (CH_3_) ethyl group	1468	1466	C=C stretching	1611	1611
Ring def.	1230	1233	C=C benzene stretching	1476	1472
Ring def.	992	996	In-plane pyrrole stretching	1332	1337
ρ_r_ (C_2_H_5_)	924	925	C-H bending	1288, 1164, 1120	1289, 1163, 1120
π (ring)	755	752	In plane C-H deformation	754	754
C≡N stretching bands of TCNQ	2214, 1673	2215, 1671	C≡N stretching bands of TCNQ	2219, 1673	2221, 1676
C=C-H bending of TCNQ	1611	1615	C=C-H bending of TCNQ	1614	1615
C-CN stretching of TCNQ	1449	1451	C-CN stretching of TCNQ	1449	1449
C=C ring stretching of TCNQ	1209	1213	C=C ring stretching of TCNQ	1211	1207

**Table 2:** Electrical properties for glass/ITO/*MM*/Ag devices.

Device	*μ* (m^2^ V^−1^ s^−1^)	*p_0_* (m^−3^)	*P_0_* (J^−1^ m^−3^)	*N_t(e)_* (m^−3^)
**CoPc-TCNQ**	4.93 × 10^−10^	1.02 × 10^25^	9.92 × 10^44^	1.09 × 10^25^
